# Early co-occurrence of a neurologic-psychiatric disease pattern in Niemann-Pick type C disease: a retrospective Swiss cohort study

**DOI:** 10.1186/s13023-014-0176-7

**Published:** 2014-11-26

**Authors:** Lucia Abela, Barbara Plecko, Antonella Palla, Patricie Burda, Jean-Marc Nuoffer, Diana Ballhausen, Marianne Rohrbach

**Affiliations:** Division of Child Neurology, University Children’s Hospital Zurich, Steinwiesstrasse 75, 8032 Zurich, Switzerland; Children’s Research Centre, University Children’s Hospital Zurich, Zurich, Switzerland; Radiz–Rare Disease Initiative Zurich, Clinical Research Priority Program for Rare Diseases, University of Zurich, Zurich, Switzerland; Department of Neurology, University Hospital Zurich, Zurich, Switzerland; Division of Metabolism, University Children’s Hospital Zurich, Zurich, Switzerland; University Institute of Clinical Chemistry, University Children’s Hospital Bern, Bern, Switzerland; Center of Molecular Diseases, University Children’s Hospital Lausanne, Lausanne, Switzerland

**Keywords:** Niemann-Pick type C, Diagnostic delay, Period prevalence rate, NP-C suspicion index

## Abstract

**Background:**

Niemann-Pick disease type C (NP-C) is a rare autosomal recessive disorder of lysosomal cholesterol transport. The objective of this retrospective cohort study was to critically analyze the onset and time course of symptoms, and the clinical diagnostic work-up in the Swiss NP-C cohort.

**Methods:**

Clinical, biochemical and genetic data were assessed for 14 patients derived from 9 families diagnosed with NP-C between 1994 and 2013. We retrospectively evaluated diagnostic delays and period prevalence rates for neurological, psychiatric and visceral symptoms associated with NP-C disease. The NP-C suspicion index was calculated for the time of neurological disease onset and the time of diagnosis.

**Results:**

The shortest median diagnostic delay was noted for vertical supranuclear gaze palsy (2y). Ataxia, dysarthria, dysphagia, spasticity, cataplexy, seizures and cognitive decline displayed similar median diagnostic delays (4–5y). The longest median diagnostic delay was associated with hepatosplenomegaly (15y). Highest period prevalence rates were noted for ataxia, dysarthria, vertical supranuclear gaze palsy and cognitive decline. The NP-C suspicion index revealed a median score of 81 points in nine patients at the time of neurological disease onset which is highly suspicious for NP-C disease. At the time of diagnosis, the score increased to 206 points.

**Conclusion:**

A neurologic-psychiatric disease pattern represents the most characteristic clinical manifestation of NP-C and occurs early in the disease course. Visceral manifestation such as isolated hepatosplenomegaly often fails recognition and thus highlights the importance of a work-up for lysosomal storage disorders. The NP-C suspicion index emphasizes the importance of a multisystem evaluation, but seems to be weak in monosymptomatic and infantile NP-C patients.

**Electronic supplementary material:**

The online version of this article (doi:10.1186/s13023-014-0176-7) contains supplementary material, which is available to authorized users.

## Background

Niemann-Pick disease type C (NP-C) is a rare autosomal recessive disorder characterized by lysosomal cholesterol transport abnormalities. NP-C has an estimated incidence of 1:120′000 [1-4]. 95% of the NP-C patients harbor mutations in the *NPC1* gene, while only 4% carry mutations in the *NPC2* gene [5]. *NPC1* encodes a large transmembrane glycoprotein [6] and *NPC2* a smaller soluble lysosomal protein, respectively [7]. Both proteins are involved in the transport of intracellular cholesterol, that derives from endocytosed LDL particles [8]. Dysfunction of these proteins leads to impaired intracellular trafficking and accumulation of unesterified cholesterol in lysosomes and late endosomes [9,10]. The lipid storage pattern differs markedly in visceral and neural tissues [11-14]. Liver and spleen mainly accumulate cholesterol and other lipid compounds, while in brain tissue accumulation of glycosphingolipids prevails [11,12,15]. Visceral storage leads to early clinical symptoms including prolonged neonatal jaundice and isolated splenomegaly with or without hepatomegaly [8]. Neuronal storage results in meganeurite formation and ectopic dendritogenesis, neuroaxonal dystrophy with demyelination and accumulation of Alzheimer’s-like neurofibrillary tangles [8,15,16]. The resulting neurological and psychiatric disease pattern is quite heterogeneous [8,16,17], even though neurodegeneration seems to follow a distinct distribution pattern, first affecting highly vulnerable neurons such as Purkinje cells in the cerebellum [15]. The NP-C suspicion index tool was developed in 2012 in order to help clinicians to better identify patients at risk for NP-C disease [18]. Three clinical categories including visceral, neurological and psychiatric symptoms are scored according to their relative association with NP-C disease. Patients with a score >70 points should be referred for further testing. In recent years, several reports have further improved the awareness for NP-C disease and shed light on its natural history [16,19-26]. With this retrospective study of the Swiss NP-C cohort, we intend to better characterize the disease manifestations in different age groups with a particular focus on neurological and psychiatric symptoms.

## Methods

### Study design

The study presented here is an observational retrospective cohort study including all known Swiss NP-C patients. Seven patients of this cohort have been included in a previous study and can be identified by their respective patient ID in the legend of Table 1. Clinical data were collected by retrospective analysis of all available medical records, in particular visceral, neurological and psychiatric symptoms. The observation period included data from the onset of the first clinical symptom and ended in September of 2013. NP-C disease was confirmed by either biochemical (filipin staining and cholesterol esterification) and/or molecular analysis. Patients were characterized into early-infantile (0–2 years), late-infantile (2–6 years), juvenile (6–15 years) and adult (>15 years), according to the age of neurological onset [4]. Period prevalence rates of clinical symptoms, i.e. visceral, neurological or psychiatric, were calculated in age groups of 5 to 20 years intervals, respectively. The period prevalence rates of respective symptoms among our cohort were calculated by the number of affected patients divided by the total number of patients alive in this age period. Data are presented in median ±2 standard deviations and the corresponding range. The NP-C suspicion Index published in 2012 [18] was assessed retrospectively for the time of neurological disease onset and for the time of diagnosis. Scores at the time of neurological disease onset are based on the information available from the chart reports. For index patients, the delay to diagnosis was defined as the time period between first recorded visceral, neurological or psychiatric symptoms compatible with NP-C and the time of diagnosis (biochemically and/or genetically defined). The study was conducted in accordance with an approval of the Local Ethics Committees granted to our institution and valid until 31^th^ of December 2013.

**Table 1:** **Summary of clinical, biochemical and genetic patient data (n = 14)**

**Patient**	**Sex**	**Age at diagnosis**	**Age at start of treatment**	**New symptoms under therapy**	**Age at last visit**	**Subtype**	**Chitotriosidase**	**Filipin staining**	**Cholesterol esterification**	**Bone marrow**	**Genetics (** ***NPC1*** **)**
1a*	m	30	31	-	33	Juvenile	Normal	Clearly abnormal	na	Sea blue histiocytes, foam cells	p.Arg978Cys/delX1-X6
1b*	f	27	28	Mild Polyneuropathy	30	Adult	9.9 mU/ml (0.0–4.6)	Clearly abnormal	na	na	p.Arg978Cys/delX1-X6
1c*	f	22	22	-	24	Adult	Normal	Clearly abnormal	na	na	p.Arg978Cys/delX1-X6
2a*	f	15	30 (− 32)	-	^†^32	Juvenile	8.2 mU/ml (0.0–4.6)	Clearly abnormal	Abnormal	Sea blue histiocytes, foam cells	p.Pro474Leu/p.Ile1094Thr
2b*	m	19	29	-	35	Juvenile	Normal	Clearly abnormal	na	Sea blue histiocytes, foam cells	p.Pro474Leu/p.Ile1094Thr
**3a**	m	15	-	-	15	Juvenile	Normal	Variant	na	na	p.Ser940Leu/p.Pro1007Ala
**3b**	f	10	-	-	10	Juvenile	Normal	na	na	na	p.Ser940Leu/p.Pro1007Ala
4a*	m	18	29	Dysphagia	29	Juvenile	11.3 mU/ml (0.3–3.7)	Clearly abnormal	na	Sea blue histiocytes, foam cells	p.Ile1061Thr/p.Ile1061Thr
4b*	m	16	27	Dysphagia, dystonia, seizures	27	Juvenile	4.4 mU/ml (0.3–3.7)	Clearly abnormal	na	Sea blue histiocytes, foam cells	p.Ile1061Thr/p.Ile1061Thr
**5**	m	2	3	VSGP	5	Late-infantile	24.5 mU/ml (0.0–1.03)	Variant	na	Sea blue histiocytes, foam cells	p.Ile1067Thr/p.Asp948Asn
**6**	m	7.5	-	-	^†^7.5	Late-infantile	na	Clearly abnormal	na	Foam cells	c.1554-1009G > A (intron 9)/p.Tyr1081*
**7**	f	50	50	Dysphagia	51	Adult	Normal	Clearly abnormal	na	Sea blue histiocytes, foam cells	p.Asp898Asn/ p.Pro1245Cysfs*12
**8**	f	23	24	-	25	Adult	Normal	Clearly abnormal	na	na	na
**9**	f	15	-	-	^†^25	Juvenile	na	Clearly abnormal	Abnormal	na	na

na: not available; ^†^deceased, *Patients included in [23]: 1a > 044, 1b > 042, 1c > 043, 2a > 020, 2b > 021, 4a > 018, 4b > 019.

### Biochemical and molecular analysis

Bone marrow histology and measurement of chitotriosidase activity in plasma were performed as described and included when available [27]. Filipin staining of cultured fibroblasts was performed and interpreted as previously described [28]. Cholesterol esterification assays were performed by M. Vanier in two patients [28]. Molecular analysis of *NPC1* and *NPC2* was performed in the Institute of Human Genetics in Heidelberg (Germany) and in the Center for Molecular Diseases in the University Hospital in Lausanne (Switzerland).

### Ethical approval and consent

The study was conducted in accordance with an approval of the Local Ethics Committees granted to our institution and valid until 31^th^ of December 2013. This approval allows treating physicians to perform retrospective data evaluation without prior informed consent.

## Results

Between 1994 and 2013, 14 patients derived from nine non-consanguineous families were diagnosed with NP-C. Two patients had late-infantile, eight patients a juvenile and four patients an adult-onset form of NP-C disease. Table 1 provides a detailed overview on clinical, biochemical and genetic findings as well as age of treatment initiation with Miglustat. Additional file 1: Table S1 provides individual information on clinical symptoms at neurological disease onset and at the time of diagnosis.

### Biochemical and genetic analysis

Filipin staining of cultured fibroblasts was clearly abnormal in 11 patients, variant in two and unavailable in one patient. Plasma chitotriosidase activity was elevated in five patients, normal in seven and unavailable in two patients. Histology of bone marrow was available for eight patients, all of whom showed sea blue histiocytes and/or foam cells. Molecular analysis confirmed the diagnosis in family 1, 2, 3 and 4 and in patient 5, 6 and 7. Patient 8 was diagnosed by positive filipin staining only. Patient 9 was diagnosed by abnormal cholesterol esterification and abnormal filipin staining.

### Clinical symptoms

#### Neurological symptoms

**Figure 1:**
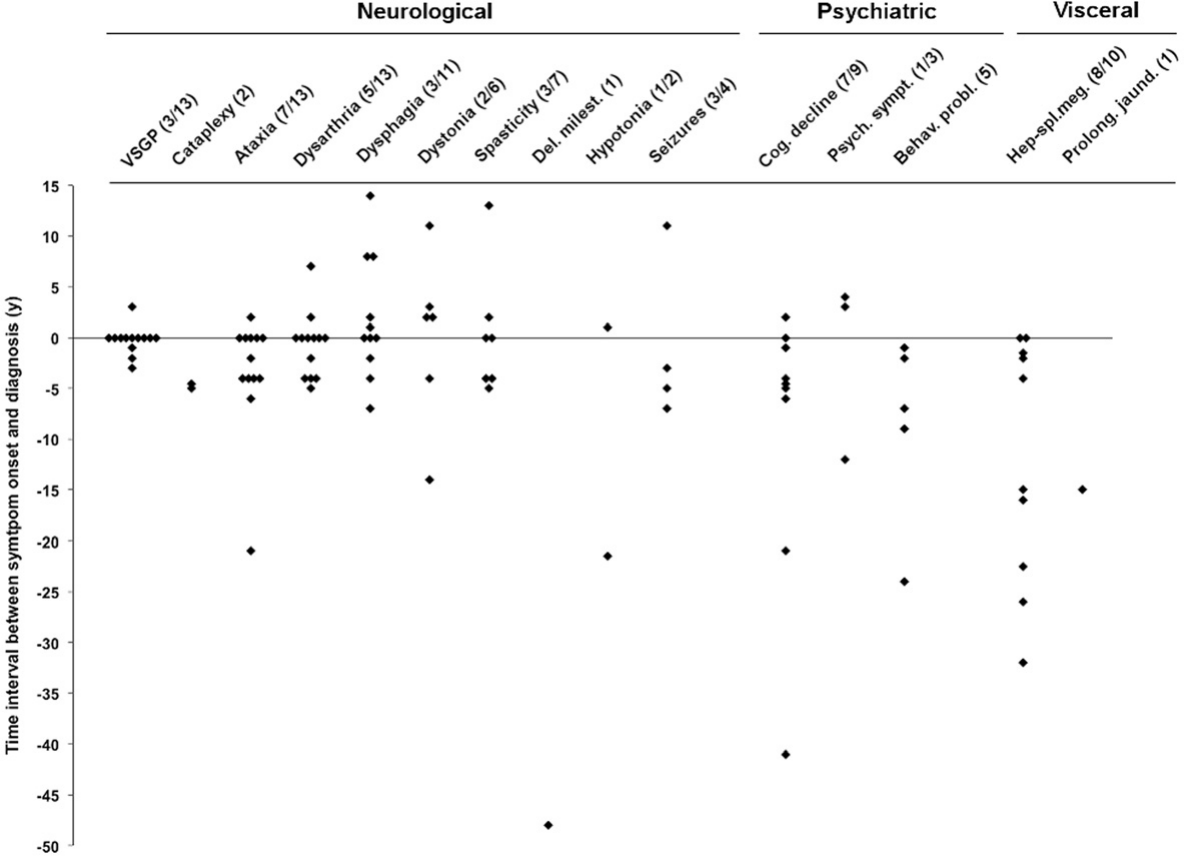
**Neurological, psychiatric and visceral symptom onset in relation to diagnosis.** Time interval from the onset of first recorded neurological, psychiatric and visceral symptoms to the diagnosis. Time at diagnosis = 0. The first number in brackets represents patients with symptom onset before diagnosis and includes index patients only. The second number represents the total patient number. Abbreviations: Del. milest. = delayed milestones; Cog. decline = cognitive decline; Psych. symptoms = psychotic symptoms; Behav. probl. = behavioural problems; Hep-spl.meg. = Hepatosplenomegaly; Prolong. jaund. = prolonged jaundice.

Documentation of neurological symptoms prior to diagnosis was available in nine patients only (1a, 2a/b, 3a, 4a/b, 6, 7, 8) with onset of symptoms ranging from of three to 46 years. In this group, the median diagnostic delay from the onset of the first recorded neurological symptom to the diagnosis was 5 ± 12.4, range 4–21 years. Presenting neurological symptoms included ataxia in six, dysarthria in two, spasticity in two, dystonia in one, cataplexy in one, vertical nuclear gaze palsy (VSGP) in one and seizures in one of nine patients. Three patients displayed a combination of neurological symptoms. At the time of diagnosis, ataxia was present in 13, VSGP in 12, dysarthria in 11, spasticity in five, dystonia in two and cataplexy in two of 14 patients. Median diagnostic delays of individual neurological symptoms were calculated for index patients only (Figure 1). The shortest median diagnostic delay is seen with VSGP (2 ± 2.0, range 1–3 years) while dysphagia (4 ± 5, range 2–7 years), seizures (5 ± 4, range 3–7 years), ataxia (4 ± 13.1, range 2–21 years), dysarthria (4 ± 2.2, range 2–7 years), spasticity (4 ± 1.2, range 4–5 years) and cataplexy (4.8 ± 0.7, range 4.5–5 years) have similar medians of diagnostic delay. Dystonia displays the longest diagnostic delay (9 ± 14.1, range 4–14 years), but was present only in two patients prior to diagnosis.

#### Psychiatric symptoms

Four patients (1a, 3a, 4b, 9) exhibited behavioural disturbances before diagnosis: aggressive behaviour in patient 1a from the age of six years, social withdrawal and emotional lability in patient 3a from the age of nine years, emotional lability and absence-like episodes in patient 4b from the age of seven years and emotional lability in patient 9 from the age of 14 years. Behavioural disturbances had a median diagnostic lag of 7 ± 18.5, range 1–24 years (Figure 1). Out of these patients with documented neurological onset prior to diagnosis, cognitive decline was reported before or at the onset of neurological symptoms in six of nine patients. At the time of diagnosis, cognitive decline was reported in 12 of 14 patients. The median diagnostic delay of cognitive decline was 5 ± 28.8, range 1–41 years (Figure 1). Patient 1a exhibited psychotic episodes after the onset of neurological symptoms but prior to diagnosis. Cognitive decline and recurrent depressive/psychotic episodes were presenting symptoms in patient 1b.

#### Visceral symptoms

Patient 8 and 9 presented with neonatal onset hepatosplenomegaly accompanied by prolonged neonatal jaundice in the latter. Four patients (1a, 4a, 4b, 5) exhibited splenomegaly during infancy at the age of four years, two years, three years as well as six months, respectively. Patient 7 underwent splenectomy at the age of 18 years. Electron-microscopic analysis of spleen tissue revealed sea blue histiocytes. The diagnosis of Niemann-Pick was suspected. Additional biochemical analysis showed slightly reduced activity of sphingomyelinase in fibroblasts, however, not in the range to diagnose Niemann-Pick type A/B. Patient 7 was not further seen by specialists and not diagnosed with NP-C until 32 years later. In patient 1b, 3a and 3b splenomegaly had only been recognized at the time of diagnosis. Aside from patient 5 who was diagnosed with NP-C disease at the age of two years based on his visceral manifestation, the remaining patients (1a, 4a, 4b, 8, 9) were diagnosed only when neurological symptoms emerged. In those with previously known (hepato-)splenomegaly, the median diagnostic delay was 15.5 ± 23.2, range 1.5–32 years (Figure 1).

### Intrafamilial heterogeneity

To highlight the intrafamilial heterogeneity, we subsequently provide details on disease onset and the clinical course of families with several affected siblings.

#### Family 1

Patient 1a presented with ataxia and cognitive decline from the age of 9 years. He developed dystonia and dystonic-athetoid movement disorder in the second decade and suffered dysphagia, rare psychotic episodes and seizures in the third decade, respectively. He was investigated for Chorea Huntington, Morbus Wilson, syphilis, neuroborreliosis, HIV, systemic lupus erythematodus, Vitamin B12 and folinic acid deficiency and thyroid disorders before the diagnosis of NP-C was made at the age of 30 years. Patient 1b had suffered from sensorineural hearing loss from the age of 9 years. In the late second decade, she developed cognitive decline. She presented with several depressive and psychotic episodes from the age of 24 years and was diagnosed with mild ataxia and dysarthria in the third decade. She was tested for subacute sclerosing panencephalitis, syphilis, HIV, cerebrotendinous xanthomatosis and mitochondrial cytopathies before she was diagnosed with NP-C at the age of 27 years. Patient 1c was only diagnosed by pedigree analysis at the age of 22 years. She suffered progressive hearing loss from the age of 18 years. During adolescence she had exhibited very mild cognitive decline and was found with VSGP with delayed vertical saccades at the time of diagnosis.

#### Family 2

Patient 2a presented with ataxia, spasticity, VSGP and cognitive decline at the age of 12 years. She was investigated for metachromatic leukodystrophy before she was diagnosed with NP-C at the age of 15 years. In the third decade, she experienced a rapid progression with severe dysarthria, dysphagia, ataxia, tetraspasticity and hyperkinetic movement disorder. She deceased at the age of 32 years. Patient 2b displayed VSGP, dysarthria and cognitive decline at the age of 19 years. In the third decade, he exhibited a slowly progressing course with ataxia, dysphagia, a first psychotic episode and mild demyelinating polyneuropathy.

#### Family 3

Patient 3a suffered from an early disease onset with cataplexy and cognitive decline from the age of nine years. In the second decade, he showed a progressive course with VSGP, ataxia, dysphagia, seizures and dementia. Before the diagnosis of NP-C was made at the age of 15 years, he was investigated for adrenoleukodystrophy, metachromatic leukodystrophy and Gaucher disease. Patient 3b was only diagnosed by pedigree analysis at the age of ten years. She exhibited splenomegaly, VSGP, ataxia and mild cognitive decline until the last clinical visit at the age of 11 years.

#### Family 4

Patient 4a and 4b were found to have splenomegaly at the age of 2 and 3 years. Patient 4b was misdiagnosed with Gaucher disease at the age of five and a half years. Treatment for Gaucher disease was not started due to isolated hepatosplenomegaly. Both patients were re-evaluated due to ataxia, spasticity and VSGP and correctly diagnosed with NP-C at the age of 16 and 18 years, respectively. In the third decade, patient 4a exhibited mild spasticity, dysphagia and mild cognitive decline, while patient 4b showed a progressive course with dystonia, dysphagia, spasticity, psychotic episodes, seizures and cognitive decline.

### Period prevalence rates of clinical symptoms in the study cohort

**Figure 2:**
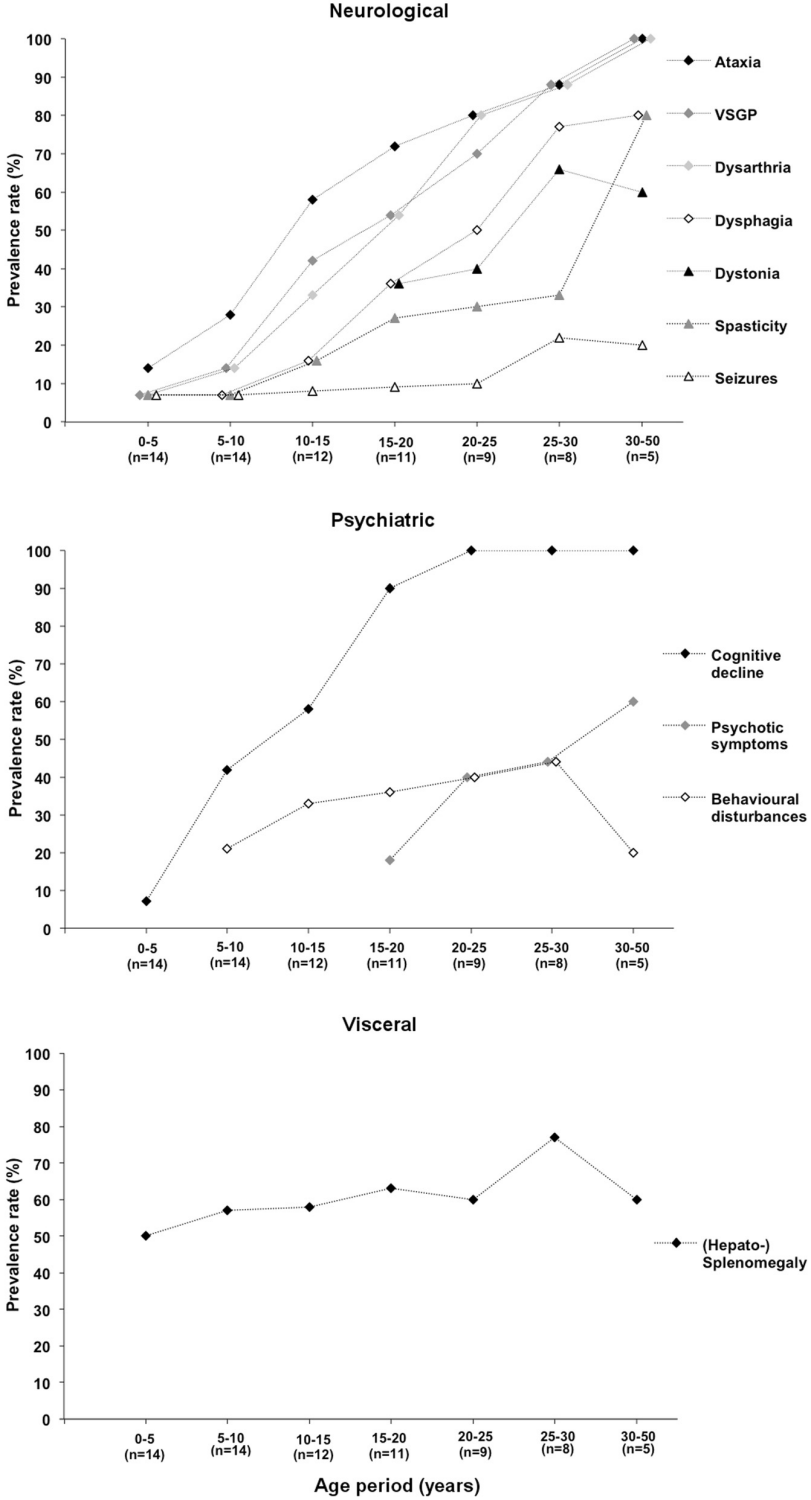
**Presentation of clinical symptoms in terms of period prevalence rates in the study cohort.** Age periods are represented in 5 to 20 years intervals on the x-axis. The period prevalence rates (%) were calculated for each clinical symptom by the number of affected patients in the respective age period divided by the total number of patients alive in this period. The total patient number is represented in brackets for each age period on the x-axis.

Figure 2 illustrates the period prevalence rates of neurological, psychiatric and visceral symptoms for the respective age periods in our cohort. Developmental milestones, hypotonia and cataplexy have been excluded from the neurological part and prolonged jaundice from the visceral part of the figure, respectively. These symptoms occurred in only one or two of our patients in early childhood (Additional file 1: Table S1) and did not show a longtime evolution. Decreases in the period prevalence rates of dystonia, behavioural disturbances and hepatosplenomegaly are caused by either death or dropouts due to age. Information on the beginning of treatment is not considered in this figure. Five patients presented new symptoms under treatment (Table 1), none of the existing symptoms were reversible under treatment.

### NP-C suspicion index

**Figure 3:**
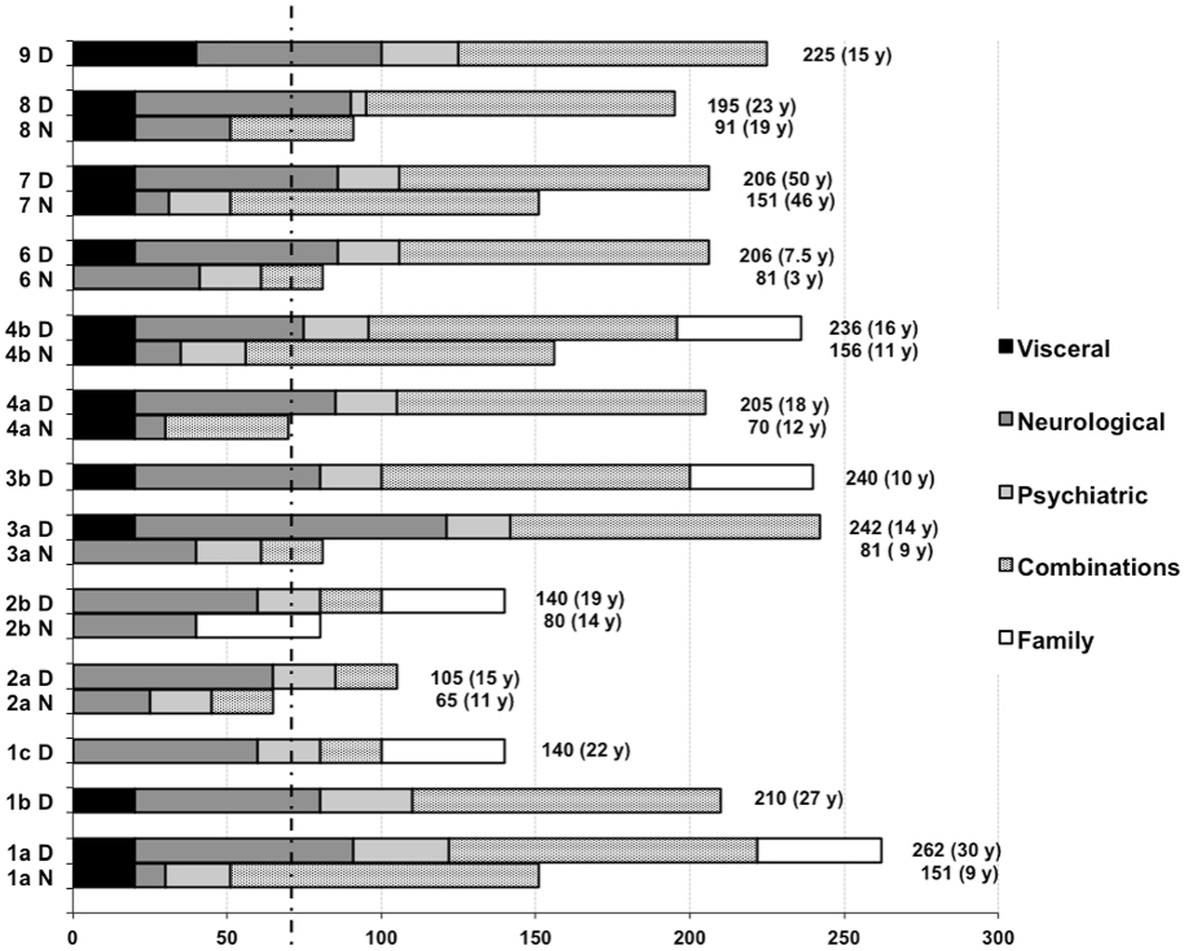
**Comparison of the NP-C suspicion index at neurological disease onset (N) and at diagnosis (D).** The NP-C suspicion index score was calculated in nine patients at the time of documented neurological disease onset (N) and in 13 patients at the time of diagnosis (D). The dotted line represents the cut-off of 70 points indicating high suspicion for NP-C disease. Combination categories include neurological/psychiatric, visceral/neurological and visceral/psychiatric scores.

Figure 3 demonstrates the NP-C suspicion index score at the time of neurological disease onset (N) and at the time of diagnosis (D). The median score at neurological disease onset in nine patients (including affected siblings) was 81 ± 76.2, range 65–156 points. All of these patients had a highly suspicious score (≥70 points). At the time of diagnosis, the median score was 206 ± 92, range 105–262 points in 13 patients. Patient 5 was excluded from indexing as the neurological disease onset was after diagnosis. In four patients (patient 1b, 1c, 3b, 9), it was not possible to evaluate the onset of neurological symptoms based on the medical records available.

## Discussion

Diagnosis of NP-C disease still poses a considerable challenge to clinicians, hampered by the lack of specific biomarkers and a remarkable inter- and intrafamilial heterogeneity. In order to critically analyze the onset of symptoms and clinical diagnostic work-up of NP-C disease, we retrospectively evaluated the Swiss cohort of 14 patients from nine families that have been diagnosed with NP-C between 1994 and 2013. 12 patients had mutations of the *NP-C1* gene, while in two patients the diagnosis of NP-C rested on abnormal filipin staining and cholesterol esterification. Genotype-phenotype correlation is limited in our group, as most of the patients that underwent genetic analysis were compound heterozygous. Intrafamilial heterogeneity was marked in family 1 in terms of onset and characteristics of symptoms. The other families described here differed from each other in the course of progression. Visceral symptoms preceded neurologic or psychiatric symptoms in six patients irrespective of the age of onset. In two juvenile and one adult patient, visceral symptoms were only recognized at the time of diagnosis, which is in line with published data in adult patients [20,22]. This stresses the importance of including lysosomal storage diseases in the work-up of isolated hepatosplenomegaly. In addition, it emphasizes the importance of searching for organomegaly in neurologic or psychiatric disorders of unclear etiology [18]. Psychiatric symptoms preceded neurological onset in the form of cognitive decline in six and behavioural problems in four of those nine patients where documentation provided exact age at neurological disease onset. Nevertheless, psychiatric symptoms, though recognized, were only taken into account at the time of first neurological assessment.

Cognitive decline may be difficult to recognize in early disease stages [8]. Poor school performance, learning disabilities, expressive language disorder and attention deficit-hyperactivity have been described in late-infantile and juvenile-onset patients [8,17,21,25] while more specific symptoms such as dysexecutive syndrome, reduced processing speed and memory impairment occur later [22,26]. Data from the international NP-C disease registry show that cognitive impairment is one of the most consistent finding across all age-at-onset categories [24]. Within our patient cohort, cognitive decline was present in a total 13 of 14 patients at some point of disease.

Cerebellar and brainstem signs such as ataxia, dysarthria and VSGP were the most common and earliest neurological features in our cohort and were followed by spasticity, dystonia and dysphagia after a median disease course of five to ten years. VSGP displayed high period prevalence rates, but in nine patients was only detected at the time of diagnosis. Our findings in nine patients with verifiable neurological disease onset show, that NP-C most commonly manifests with neurologic-psychiatric disease pattern (4/9 patients) and a neurological-psychiatric-visceral pattern (4/9 patients) followed by a visceral-neurological pattern (1/9 patients). Those findings are in line with the existing natural history studies [19,21,24,25]. A retrospective evaluation of a cohort of 38 patients revealed that cognitive and coordination deficits are early disease indicators thus supporting our findings [23]. Nevertheless, a neurological-psychiatric disease pattern is not specific for NP-C disease.

Among our cohort, visceral symptoms had the highest median diagnostic delay (15.5 ± 23.2 years). Cerebellar and brainstem symptoms such as ataxia (4 ± 13.1 years), dysarthria (4 ± 2.2 years) and dysphagia (4 ± 5 years) had median diagnostic delays similar to cognitive decline (5 ± 28.8 years). The same delays were noted for the rarer symptoms cataplexy (4.8 ± 0.7 years), seizures (5 ± 4 years) and spasticity (4 ± 1.2 years). The shortest median diagnostic delay was observed for VSGP (2 ± 2.0 years), indicating that this symptom may be more easily associated with NP-C disease. To assess the diagnostic impact of the NP-C prediction score, we retrospectively calculated the score at the time of diagnosis and found a median score of 206 ± 92 points. At time of neurological disease onset, retrospective calculation gave an estimate of 81 ± 76.2 points (>70 points suggesting high suspicion), but for these patients, the median diagnostic delay was still 5 ± 12.4 years. This is in line with a larger survey from the international NP-C disease registry [24]. The NP-C suspicion index tool was recently analyzed by ROC analysis [29], showing that visceral manifestation had the weakest and neurological symptoms the highest discriminatory power in juvenile and adolescent patients. In infantile patients (<4 years), the discriminatory performance was very poor. To overcome these problems, a revised NP-C suspicion index with an improved predictive ability and an index for paediatric patients have been developed recently [30,31].

## Conclusion

Thus, especially in infantile patients, a work-up of isolated hepatosplenomegaly has to include lysosomal storage disorders such as Gaucher disease but also NP-C. In juvenile and adolescent patients, we emphasize that co-occurrence of neurological symptoms and cognitive decline occurs early.

### Limitations

This study is based on careful extraction of all available data in the patient charts. This might constitute a potential weakness of the current study.

## Additional file

Additional file 1: Table S1. Overview on neurological, visceral and psychiatric symptoms at age of neurological disease onset (N) and at age of diagnosis (D).

## Abbreviations

NP-C: Niemann-Pick type C
VSGP: Vertical supranuclear gaze palsy
