# Supplementary material for: Early co-occurrence of a neurologic-psychiatric disease pattern in Niemann-Pick type C disease: a retrospective Swiss cohort study
Source: Orphanet J Rare Dis. 2014 Nov 26;9:176. doi: 10.1186/s13023-014-0176-7 (PMC4253629; doi:10.1186/s13023-014-0176-7)
Supplement: Additional file 1: Table S1. — Overview on neurological, visceral and psychiatric symptoms at age of neurological disease onset (N) and at age of diagnosis (D). [file 13023_2014_176_MOESM1_ESM.docx]

| **Additional file Table S1. Overview on neurological, visceral and psychiatric symptoms at age of neurological disease onset (N) and at age of diagnosis (D).** | | | | | | | | | | | | | | | | | | | | | | | |
| --- | --- | --- | --- | --- | --- | --- | --- | --- | --- | --- | --- | --- | --- | --- | --- | --- | --- | --- | --- | --- | --- | --- | --- |
| **Patients** | **P 1a** | | **P 1b** | **P 1c** | **P 2a** | | **P 2b** | | **P 3a** | | **P 3b** | **P 4a** | | **P 4b** | | **P 5** | **P 6** | | **P 7** | | **P 8** | | **P 9** |
|  | **N** | **D** | **N/D** | **N/D** | **N** | **D** | **N** | **D** | **N** | **D** | **N/D** | **N** | **D** | **N** | **D** | **D** | **N** | **D** | **N** | **D** | **N** | **D** | **N/D** |
| **Age (years)** | **9** | **30** | **27** | **22** | **11** | **15** | **14** | **19** | **9** | **14** | **10** | **12** | **18** | **11** | **16** | **2** | **3** | **7.5** | **46** | **50** | **19** | **23** | **15** |
| **VSGP** |  | + | + | + |  | + | + | + |  | + | + |  | + |  | + |  |  |  |  | + |  | + | + |
| **Cataplexy** |  |  |  |  |  |  |  |  | + | + |  |  |  |  |  |  | + | + |  |  |  |  |  |
| **Ataxia** | + | + | + | + | + | + |  | + |  | + | + | + | + | + | + |  |  | + | + | + | + | + | + |
| **Dysarthria** |  | + |  | + | + | + |  | + |  | + | + |  | + |  |  |  |  | + |  | + | + | + | + |
| **Dysphagia** |  | + | + |  |  | + |  |  |  | + |  |  |  |  |  |  |  | + |  |  | + | + |  |
| **Dystonia** |  | + |  |  |  |  |  |  |  |  |  |  |  |  |  |  |  |  |  |  | + | + |  |
| **Spasticity** |  |  |  |  | + | + |  |  |  |  |  |  | + | + | + |  |  | + |  | + |  |  |  |
| **Hypotonia** |  |  |  |  |  |  |  |  |  |  |  |  |  |  |  |  |  |  |  |  | (+) | (+) |  |
| **Delayed milestones** |  |  |  |  |  |  |  |  |  |  |  |  |  |  |  |  |  |  | (+) | (+) |  |  |  |
| **Seizure** |  | + |  |  |  |  |  |  |  | + |  |  |  |  |  |  | + | + |  |  |  |  |  |
| **Prolonged neonatal jaundice** |  |  |  |  |  |  |  |  |  |  |  |  |  |  |  |  |  |  |  |  |  |  | (+) |
| **(Hepato-) Splenomegaly** | + | + | + |  |  |  |  |  |  | + | + | + | + | + | + | + |  | + | (+) | (+) | + | + | + |
| **Cognitive decline** | + | + | + | + | + | + |  | + | + | + | + |  | + | + | + |  | + | + | + | + |  |  | + |
| **Psychotic symptoms** |  | + | + |  |  |  |  |  |  |  |  |  |  |  |  |  |  |  |  |  |  |  |  |
| **Behavioural disturbances** | + | + |  |  |  |  |  |  | + | + |  |  |  | + | + |  |  |  |  |  |  |  | + |
| VSGP; vertical supranuclear gaze palsy. | | | | | | | | | | | | | | | | | | | | | | | |
